# Hydrogen Sulfide: A Gaseous Molecule in Postharvest Freshness

**DOI:** 10.3389/fpls.2018.01172

**Published:** 2018-08-27

**Authors:** Jianqiang Huo, Dengjing Huang, Jing Zhang, Hua Fang, Bo Wang, Chunlei Wang, Weibiao Liao

**Affiliations:** College of Horticulture, Gansu Agricultural University, Lanzhou, China

**Keywords:** hydrogen sulfide (H_2_S), chilling injury, antioxidant system, antifungal, interactions, senescence-related genes

## Abstract

Hydrogen sulfide (H_2_S), as a signaling molecule, is involved in the regulation of growth and development in plants. Recent studies have indicated that H_2_S also plays important roles in regulating postharvest senescence of horticultural products. The focus of this review is to summarize the synthesis of H_2_S in plants and its potential roles in alleviating the senescence of cut flowers, fruits, and vegetables during postharvest storage. During postharvest of horticultural products, H_2_S could scavenge reactive oxygen species via promoting the activity of antioxidant enzymes, thereby, sustaining the integrity of the membrane. In fruits, H_2_S effectively enhanced the tolerance of chilling by increasing the content of proline and polyphenol compounds. During postharvest storage of perishable fruits and vegetables, H_2_S significantly alleviated decay, which was caused by fungi by inhibiting the growth of fungi spores. Moreover, H_2_S interacted with other molecules synergistically (NO) or antagonistically (ethylene) to alleviate senescence of horticultural products. At the transcriptional level, H_2_S regulated the expression of senescence-related genes, which were related to degradation of proteins and chlorophyll, to delay the senescence of horticultural products. Thus, H_2_S does not only possess positive antioxidant and antifungal properties, but also significantly regulates the senescence-related gene during postharvest of horticultural products. Future studies of H_2_S in postharvest storage should focus on its molecular mechanism in the posttranslational modifications of proteins as well as its safety attributes in treated fruits and vegetables.

## Introduction

Hydrogen sulfide (H_2_S), a colorless gas with an odor of rotten eggs, is a new gaseous signaling molecule that regulates physiological processes in plants (Li et al., 2016b). Recently, many investigations have suggested that H_2_S is involved in various growth and development processes in plants including germination (Liu and Lal, 2015), lateral and adventitious root formation (Lin et al., 2012; Jia et al., 2015), stomatal movement (Scuffi et al., 2014; Papanatsiou et al., 2015; Jin and Pei, 2016), and photosynthesis (Duan et al., 2015). In addition, the roles of H_2_S in regulating plant responses to abiotic stress including drought stress (Chen et al., 2016; Jin et al., 2017), osmotic stress (Khan et al., 2017), salt stress (Lai et al., 2014), chilling stress (Fu et al., 2013), and heavy metal stress (Ali et al., 2015; Fang et al., 2017) were reported. Under abiotic stress, some researchers indicated that H_2_S could ameliorate the quality and nutrients of horticultural products such as *Capsicum annuum* L. (Kaya et al., 2018), *Brassica napus* L. (Qian et al., 2014), and *Brassica pekinensis* (Lour.) Rupr. cv. Kasumi F1 (Reich et al., 2016). In addition, a number of studies showed that H_2_S played an important role in delaying senescence of horticultural products during postharvest storage with examples in kiwifruits (Zhu et al., 2014), daylily (Liu et al., 2017), and cut flowers (Zhang et al., 2011). Recently, there were reviews that discussed H_2_S as a signal molecule or a versatile regulator in plant response to abiotic stress (Guo et al., 2016; Li et al., 2016b). However, its role in the postharvest life of horticultural products has not yet been summarized in a published review. Therefore, the main aim of this review is to summarize the importance roles of H_2_S in the postharvest life of horticultural products. This review concentrates on the mechanisms by which H_2_S is involved in maintaining the postharvest freshness such as by regulating antioxidant system, enhancing chilling tolerance, and inhibiting fungi growth. The interaction of H_2_S with other molecules and the regulation of senescence-related genes during postharvest storage process have also been discussed.

## Generation of H_2_S in Plants

**FIGURE 1:**
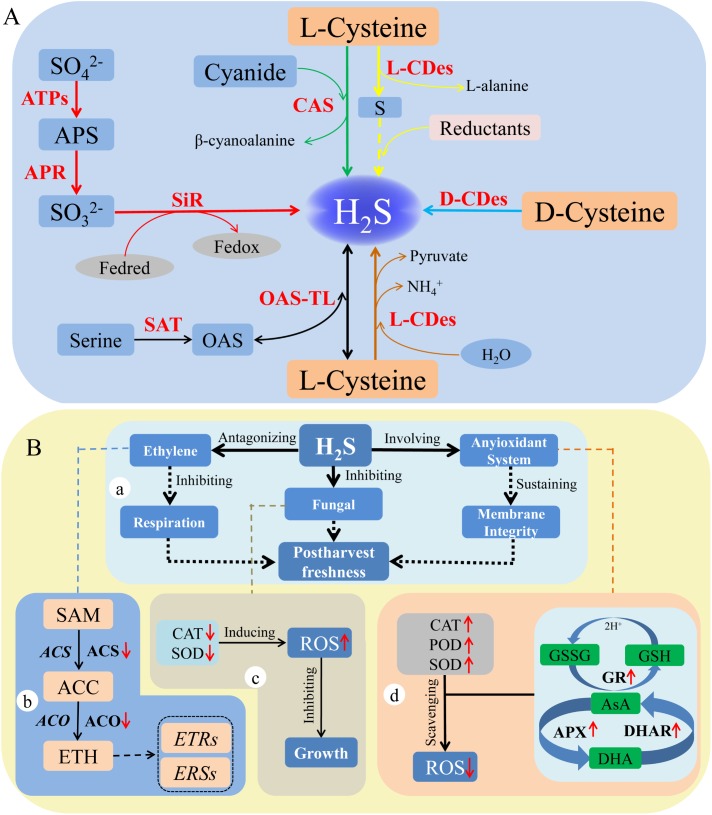
Production and function of hydrogen sulfide in plants. Production **(A)**: The red arrows indicate sulfite is reduced to H_2_S (Takahashi et al., 2011); the green arrows indicate H_2_S is produced from L-CDes catalytic pathway (Harrington and Smith, 1980); the yellow arrows show H_2_S generation in the presence of reductants (Papenbrock et al., 2007); the light blue arrows represent H_2_S generated from the pathway catalyzed by D-CDes (Riemenschneider et al., 2005); the brown arrows show the H_2_S was generated from the reaction of L-cysteine and cyanide (Hatzfeld et al., 2000); the black arrows show H_2_S is produced from the reverse reaction of synthesis of cysteine (Li, 2015). The red words represent the key enzymes involving in various pathways. Function **(B)**: a, the roles of H_2_S in delaying senescence and decay of horticultural products. b, the involvement of H_2_S in the generation and signaling of transduction of ethylene. c, the effect of H_2_S on antioxidant enzymes *in vivo* the fungi. d, the roles of H_2_S in regulating the antioxidant system in horticultural plants.

In higher plants, H_2_S emission was first observed by DeCormis in 1968 (Rennenberg, 1989). Subsequently, Wilson et al. (1978) found that many higher plants including pumpkin, cucumber, cantaloupe, corn, and soybean could release H_2_S dependent on light. The emission of H_2_S in leaf tissue of cucumber was reported by Sekiya et al. (1982). Until now, there have been six pathways through which H_2_S is generated in higher plants, and these could be summarized as follows: plants reduce sulfate to sulfide and incorporate it into organic metabolites. Before reduction, sulfate is activated by adenylation to form adenosine 50-phosphosulfate (APS), which is catalyzed by ATP sulfurylase (ATPS). In the plastids, APS is first reduced to sulfite by APS reductase (APR). Then, sulfite is further reduced to sulfide (including H_2_S) by ferredoxin-dependent sulfite reductase (SiR) (Takahashi et al., 2011; **Figure 1A**). Finally, sulfide is incorporated into the amino acid skeleton of *O*-acetylserine (OAS) by *O*-acetylserine thiol lyase (OAS-TL) to form cysteine, and its reverse reaction could release H_2_S (Li, 2015). Among these, OAS is a product from serine, which is catalyzed by serine acetyltransferase (SAT). Harrington and Smith (1980) first reported that the degradation of L-cysteine yielded H_2_S, pyruvate, and NH_4_^+^ in tobacco cells. Subsequently, some researchers found that the L-cysteine was catalyzed by L-cysteine desulfhydrase (L-CDes) to form H_2_S in other higher plants (Sekiya et al., 1982; Rennenberg, 1983; Xie et al., 2013). In addition, L-CDes also catalyzed the formation of L-alanine and elemental sulfur from L-cysteine. Then, the elemental sulfur could be reduced to H_2_S if a reductant was present (Papenbrock et al., 2007; **Figure 1A**). Two genes, *AtNFS1* and *AtNFS2*, which encoded NifS-like cysteine desulfurase have been identified in *Arabidopsis*, catalyzing cysteine to form H_2_S (Léon et al., 2002). Whereas, Nagasawa et al. (1985, 1988) indicated that D-cysteine was decomposed into H_2_S by D-cysteine desulfhydrase (D-CDes) in *Escherichia coli* and *Pseudomonas putida*. The D-CDes was isolated from *Arabidopsis thaliana* and it could catalyze D-cysteine to release H_2_S (Riemenschneider et al., 2005). However, L-CDes and D-CDes were different in substrates, enzymatic inhibitors, and subcellular localization (Guo et al., 2016). Besides, the β-cyanoalanine synthase (CAS) catalyzed L-cysteine and cyanide to form H_2_S and β-cyanoalanine (Hatzfeld et al., 2000). Jost et al. (2000) found the three genes (*CYS-C1, CYS-D1*, and *CYS-D2*), which encoded CAS in *Arabidopsis* (**Figure 1A**). Therefore, H_2_S was released from L-cysteine in the presence of hydrogen cyanide, which was catalyzed by CAS in plants. The H_2_S, especially endogenous H_2_S, which acts as a signaling molecule, can move freely from one plant cell to another. Thus, endogenous H_2_S may play important roles in plants.

## Regulatory Roles of H_2_S in Antioxidant System During Harvested Storage

Reactive oxygen species (ROS) caused by oxidative damage could accelerate senescence and ripening of horticultural products during postharvest storage. Recent investigations suggested that H_2_S could delay the senescence and ripening of horticultural products by alleviating the oxidative damage caused during postharvest storage. During postharvest of cut flowers, exogenous H_2_S could delay senescence of cut flowers by enhancing the activity of antioxidant enzymes (**Figure 1B**). For example, Zhang et al. (2011) found that a lower level of hydrogen peroxide (H_2_O_2_) and superoxide anion (O_2_^-^) in cut *Erigeron annuus* (L.) and *Salix matsudana* Koidz were sustained by exogenous H_2_S (**Table 1**). That is because the activities of catalase (CAT), superoxide dismutase (SOD), ascorbate peroxidase (APX), and peroxidase (POD) were increased by H_2_S. Thus, H_2_S could delay senescence of cut flowers by decreasing ROS-induced oxidant damage. During postharvest of fruits, Hu et al. (2012) indicated that H_2_S did not only improve the activities of CAT and POD, but also increased activities of APX and glutathione reductase (GR) during strawberry postharvest storage. Furthermore, H_2_S could also decrease the activity of lipoxygenase (LOX), which is responsible for catalyzing oxygenation of polyunsaturated fatty acids (Hu et al., 2012). Therefore, H_2_S played an antioxidant role in protecting tissue against the damaging effects of ROS. Studies on fresh-cut kiwifruit (Gao et al., 2013) and grape (Ni et al., 2016) were consistent with the previously mentioned results. Hu et al. (2014a) suggested that H_2_S could delay senescence of mulberry fruit by enhancing the activity of antioxidant enzymes, which could scavenge the ROS. Thus, H_2_S reduced the damage of lipid peroxidation and membrane by scavenging ROS, thereby, alleviating the senescence and ripening of fruits (**Figure 1B**). During postharvest of vegetables, Hu et al. (2015) reported that the postharvest senescence of water spinach was alleviated by H_2_S. In this process, H_2_S improved antioxidant capacity and sustained the energy status, which decreased the degree of leaf yellowing of water spinach. The redox balance in the senescence of daylily was promoted by H_2_S, which mainly reduced the overaccumulation of ROS via increasing the activity of CAT, APX, and SOD (Liu et al., 2017; **Table 1**). In addition, Sun et al. (2015) suggested that H_2_S could regulate phenolic metabolism to alleviate browning of fresh-cut lotus root slices, and the role of H_2_S in alleviating enzymatic browning was through inhibiting the activity of polyphenol oxidase (PPO) during postharvest storage. Thus, H_2_S acted as a gaseous regulator to increase the activities of antioxidant enzymes to affect the accumulation of ROS. As mentioned earlier, H_2_S alleviated the damage of lipid peroxidation and delayed the postharvest senescence of cut flowers, fruits, and vegetables. However, the detailed mechanism of H_2_S regulating the activity of antioxidant enzymes still needs further research in order to be well understood.

**Table 1:** Effects of hydrogen sulfide on postharvest senescence of horticultural products.

H_2_S-involved	Plant species	H_2_S-mediated effect	Reference
Antioxidant system	Cut flowers: *Erigeron annuus* and *Salix matsudana*	Increasing the activities of CAT, POD, and APX; Extending vase life of cut flowers	Zhang et al., 2011
	*Fragaria* ×*ananassa*	Improving the activities of CAT, POD, APX, and GR; Decreasing the activity of LOX; Prolonging postharvest shelf life of strawberry	Hu et al., 2012
	*Actinidia deliciosa*	Increasing the activities capacity; Alleviating senescence and tissue softening of kiwifruit	Gao et al., 2013
	*Nelumbo nucifera*	Regulating phenolic metabolism; Alleviating browning of fresh-cut lotus	Sun et al., 2015
	*Ipomoea aquatica*	Improving antioxidant capacity; Delaying senescence of water spinach	Hu et al., 2015
	*Vitis vinifera*	Reducing the accumulation of MDA, H_2_O_2_, and O_2_^-^; Alleviating postharvest senescence and decrease of firmness of grape	Ni et al., 2016
	*Hemerocallis fulva*	Increasing activities of antioxidant enzymes; Alleviating senescence of daylily	Liu et al., 2017
Chilling resistance	*Musa* spp.	Increasing the content of proline; Alleviating chilling injury of banana	Luo et al., 2015
	*Musa* spp.	Promoting the activity of the energy metabolism-related enzymes; Alleviating chilling development	Cao et al., 2018
	*Crataegus pinnatifida*	Regulating the activities of antioxidant enzyme; Promoting chilling injury of hawthorn	Aghdam et al., 2018
Antifungal	Fruits: *Malus domestica, Actinidia deliciosa, Pyrus bretschneideri Rehd.*	Inhibiting the colony growth of *A. niger* and *P. italicum*; Decreasing the postharvest decay of fruits	Fu et al., 2014
	*Pyrus pyrifolia*	Inhibiting the growth of *A. niger* and *P. expansum*; Prolonging the shelf life of fresh-cut pear	Hu et al., 2014b
	*Ipomoea batatas* L.	Inhibiting the growth of fungal pathogens; Inhibiting the senescence and decay of fresh-cut sweet potato	Tang et al., 2014
	*Prunus persica* L.	Inhibiting the spore germination rate, mycelial development diameter, and pathogenicity; Alleviating the brown rot of peach	Wu et al., 2018

## Involvement of H_2_S in Postharvest Chilling Stress

Cold storage is widely used to delay senescence and ripening of fruits during postharvest handling. However, storage at low non-freezing temperatures causes chilling stresses for fruits like banana (Luo et al., 2015) and peach (Cao et al., 2018), which are extremely sensitive to chilling injury. Chilling injury not only retards shelf life, but also reduces quality of fruits in the postharvest storage process. In recent years, some researchers reported that H_2_S could alleviate chilling injury, which is caused by the low temperature, during postharvest storage. Luo et al. (2015) found that H_2_S fumigation could alleviate chilling injury of harvested banana by promoting the antioxidant capacity and increasing the content of proline. During that process, the activities of antioxidant enzymes like SOD, CAT, and APX were increased by H_2_S, for these to scavenge ROS (**Table 1**). Aghdam et al. (2018) also found that H_2_S could regulate the activities of antioxidant enzymes to sustain the lower malondialdehyde (MDA), ameliorating chilling injury of hawthorn fruit. It showed that H_2_S could increase the activity of antioxidant enzymes to decrease the membrane lipid peroxidation, so as to sustain the high tolerance for chilling. In parallel, the content of polyphenol increased in H_2_S treatment, and this was because the activity of phenylalanine ammonia lyase was increased, while the activity of PPO was decreased by H_2_S (Luo et al., 2015). Furthermore, proline content was also elevated by H_2_S, which resulted from the increase of the activity of 1-pyrroline-5-carboxylate synthetase and the decrease of activity of proline dehydrogenase. Proline, as an osmotic substance, could maintain the high cytosolic concentration of the cell to increase the ice point. This indicates that H_2_S could directly or indirectly alleviate chilling injury by increasing the content of proline. Li et al. (2016a) suggested that H_2_S could improve the tolerance of banana to chilling injury by promoting the activities of the energy metabolism-related enzymes such as H^+^-ATPase, Ca^2+^-ATPase, cytochrome C oxidase, and succinate dehydrogenase. In addition, the quality of banana such as firmness was also sustained by H_2_S under chilling condition (Li et al., 2016a; **Table 1**). Thus, H_2_S seemly regulated the content of osmotic substances, sustaining the tolerance to chilling stress. Taken together, it could be seen that the tolerance to chilling injury of fruits was promoted by H_2_S, which sustained the integrity of the membrane and maintained sufficient energy via regulating activities of enzymes and content of osmotic substances.

## Inhibition of Fungal Growth by H_2_S in Postharvest Horticultural Products

Generally, decay of some perishable horticultural products happens during storage and transportation. The main reason behind decay is the activity of plant fungal pathogens, which is usually promoted by wounds. In recent years, some research has found that H_2_S, applied as a regulator or fungicide, could alleviate senescence and decay of postharvest horticultural products by inhibiting the growth of fungi. Fu et al. (2014) reported that exogenous H_2_S (0.5 mM NaHS solution) effectively decreased the decay of fruits such as apple, kiwifruit, pear, sweet orange, mandarin, and tomato by inhibiting the colony growth of *Aspergillus niger* and *Penicillium italicum*, which mainly inhibited spore germination, germ tube elongation, and mycelial growth (**Table 1**). This was possible because H_2_S induced the accumulation of ROS in *A. niger* by decreasing the activity of antioxidant enzymes including CAT and SOD (**Figure 1B**). Contrarily, the investigations in postharvest freshness of horticultural plants suggested that H_2_S could scavenge ROS accumulation by promoting the activities of CAT, SOD, APX, and GR (Zhang et al., 2011; Hu et al., 2012; Liu et al., 2017). Fu et al. (2014) also attributed the different effects of H_2_S on microbes and plants to their different tolerances to H_2_S. Hence, the low level H_2_S (0.5 mM NaHS solution) seemly induced the accumulation of ROS in fungal *in vivo* by decreasing the activities of antioxidant enzymes, thereby, inhibiting the growth of fungi during storage (**Figure 1B**). However, the higher level H_2_S generally was used as a fungicide to inhibit the growth and infection of fungi. Wu et al. (2018) demonstrated that the spore germination rate, mycelial development diameter, and pathogenicity of *Monilinia fructicola* were inhibited by H_2_S, especially 50 mM NaHS solution for 2 h, which alleviated brown rot of peach fruit, caused by *M. fructicola*. Hu et al. (2014b) found that H_2_S delayed the shelf life of fresh-cut slices of pear and alleviated rots by inhibiting the growth of *A. niger* and *Penicillium expansum* (**Table 1**). The inhibition of mycelium growth of *A. niger* and *P. expansum* on medium depended on the H_2_S concentrations, which was found totally inhibited at 2 mM NaHS treatment. Moreover, H_2_S also blocked the decrease of reducing sugar and soluble protein during postharvest storage of pear (Hu et al., 2014b). The role of H_2_S in inhibiting senescence and decay of fresh-cut sweet potato during postharvest also depended on exogenous H_2_S concentration, especially on the optimal concentration of 2 mM NaHS (Tang et al., 2014). Interestingly, H_2_S alleviated the decay of sweet potato via inhibiting the growth of fungal pathogens including *Rhizopus nigricans, Mucor rouxianus*, and *Geotrichum candidum*, which were isolated from sweet potato tissue, which was infected by black or soft rot (Tang et al., 2014; **Table 1**). Consequently, H_2_S (at the higher concentration) as a fungicide inhibited the growth and infection of fungi during postharvest storage. As mentioned earlier, the low level H_2_S may be acts as a gaseous regulator to affect the accumulation of ROS, while it appeared it played the role of a fungicide at high concentrations. Furthermore, H_2_S might simultaneously play both a regulator and fungicide role in inhibiting the growth and infection of fungi. Although H_2_S could induce the ROS accumulation *in vivo* to inhibit fungal infection, its role in regulating the content of ROS has still not been extensively reported. Therefore, the detailed mechanisms of H_2_S in the inhibition of the fungal community need to be studied.

## Interaction of H_2_S and Other Molecules in Postharvest Senescence

In recent studies, the synergistic or antagonistic roles of H_2_S with other molecules in postharvest senescence of horticultural products were reported. Zhang et al. (2014) reported the synergistic role of H_2_S and nitric oxide (NO) in alleviating decay and softening of strawberry. Cotreatment with H_2_S (0.8 mM NaHS solution) and NO (5 μM SNP solution) significantly maintained the quality (crust color and firmness) of strawberry and also inhibited its respiration rate and decay. Moreover, H_2_S was combined with NO to increase the activities of chitinase and beta-1,3-glucanase, which played vital roles in ameliorating damages caused by pathogen. The lower activities of pectin methylesterase (PME), polygalacturonase (PG), and endo-β-1,4-glucanase (EGase) that were related to softening of fruits were sustained by cotreatment with H_2_S and NO (Zhang et al., 2014). Based on these results, the authors concluded that the synergistic effect of H_2_S and NO could significantly delay senescence and alleviate decay of fruits. However, the mechanism of synergistic effect between H_2_S and NO on senescence of harvested produce has not been extensively reported. In another study, Al Ubeed et al. (2017) found that H_2_S alleviated the senescence and postharvest deterioration of pak choy by inhibiting the ethylene generation and ethylene action during storage. 1-Methylcyclopropene (1-MCP), as a competitive inhibitor of the action of ethylene, could inhibit the production of endogenous ethylene. Al Ubeed et al. (2018) found that 1-MCP and H_2_S were equally effective in inhibiting respiration and endogenous ethylene production when pak choy was stored in the absence of exogenous ethylene. H_2_S also prolonged postharvest senescence and ripening of banana during storage via antagonizing the effect of ethylene (Ge et al., 2017). It is well known that the ripening and senescence of fruits would be accelerated by the generation of ethylene. This is because ethylene accelerates respiration and other biochemical processes such as increasing cell membrane permeability and organic transformation. Thus, H_2_S, as a signaling molecule, significantly alleviates the senescence and ripening of fruits by interfering with ethylene synthesis during storage of horticultural products. Moreover, H_2_S also regulated the expression of ethylene synthesis genes and ethylene receptor genes at the transcriptional level. During ripening and senescence of kiwifruit, the expression of genes including *AdSAM, AdACS1, AdACS2, AdACO2*, and *AdACO3*, which were related to ethylene synthesis, was inhibited by H_2_S (Li et al., 2017; **Figure 1B**). However, during postharvest storage of a banana, H_2_S upregulated the expression of ethylene receptor genes such as *MaETR, MaERS1*, and *MaERS2*, which were at lower levels of expression in the presence of ethylene (Ge et al., 2017; **Figure 1B**). It has been indicated that H_2_S regulated the ethylene generation and its signal transduction at the transcriptional level. Higher levels of nutrients such as ascorbic acid, soluble protein, and reducing sugar were sustained by H_2_S during the postharvest storage of kiwifruit (Li et al., 2017). H_2_S also alleviated the yellowing and softening of banana peel, which was caused by ethylene and evidently decreased the activity of PG, which was involved in cell wall degradation (Ge et al., 2017). It can be deduced that H_2_S does not only regulates generation and signal transduction of ethylene, but also alleviates the deterioration of quality caused by ethylene of physiological parameters. To sum up, the synergistic or antagonistic effects of H_2_S with other molecules might play vital roles in the regulation of senescence and quality of harvested fruits and vegetables.

## Senescence-Related Genes Regulated by H_2_S

Postharvest senescence is a universally phenomenon for horticultural products, and this seriously affects the shelf life and quality of fruits and vegetables during storage. During senescence, the degradation of some proteins and chlorophyll occurs, and this is regulated by certain genes. The role of H_2_S in regulating senescence-related genes during postharvest storage is discussed in this review. Li et al. (2014) indicated that the relative expressions of *BoSGR, BoCLH2, BoPaO*, and *BoRCCR*, which were related to chlorophyll degradation, were downregulated by H_2_S during postharvest storage of broccoli. In the storage of broccoli, apart from *BoSGR* and *BoRCCR*, other chlorophyll degradation-related genes such as *BoNYC, BoCLH1*, and *BoPPH* were also downregulated by H_2_S (Li et al., 2015). Thus, H_2_S regulated the expression of chlorophyll degradation-related genes to inhibit degradation of chlorophyll, thereby, effectively alleviating the postharvest yellowing of broccoli. In addition, Li et al. (2015) showed that the expression of *BoCP3* (cysteine protease gene) and *BoLSC807* (aspartic protease gene) was inhibited by H_2_S, suggesting that H_2_S inhibited the degradation of proteins during the senescence process in broccoli. Meanwhile, H_2_S cloud downregulate the expression of the LOX gene, *BoLOX1*, which catalyzed the hydroperoxidation of polyunsaturated fatty acids during senescence in broccoli (Li et al., 2014). The expressions of *MdLOX2* and *MdPPO* (encoding PPO) were also downregulated by H_2_S during storage of apples (Zheng et al., 2016). It can be deduced that H_2_S delayed the senescence of fruits and vegetables by decreasing the expression of lipid peroxidation-related genes during postharvest storage. Generally, the genes that regulated the synthesis and signal transduction of ethylene were classified as senescence-related genes. Li et al. (2015) found that the expression of *BoACS2* and *BoACS3*, which were key enzymes for ethylene generation, were downregulated by H_2_S during broccoli postharvest storage. Meanwhile, the expressions of ethylene signal transduction genes, *MdERS1* (ethylene response sensor 1) and *MdETR1* (ethylene receptor 1), were also inhibited by H_2_S during the storage of apples (Zheng et al., 2016). Therefore, H_2_S could inhibit the gene expression of ethylene generation and signal transduction, thereby, alleviating the senescence of postharvest horticultural products. Taken together, H_2_S regulates the expression of senescence-related genes and delays the senescence of horticultural products during storage. However, the signaling transduction pathway of H_2_S delaying senescence of horticultural products still has not been extensively reported.

## Conclusion

Recently, a number of studies have suggested that H_2_S played a significant role in maintaining the postharvest freshness of horticultural products. Presently, available reports have suggested that H_2_S promotes postharvest life of horticultural products mainly by increasing the activity of antioxidant enzymes, alleviating chilling stress, inhibiting the growth of fungi, interacting with other molecules, and regulating senescence-related genes. Consequently, H_2_S, as a gaseous regulator or signaling molecules, may regulate various physiological and biochemical processes during postharvest storage.

For now, the role of H_2_S, in alleviating the senescence and decreasing quality, is mainly studied based on the antioxidant system and ethylene generation. However, the detailed mechanisms of H_2_S, as a gaseous regulator to inhibit the growth of fungi and regulate activities of antioxidant enzymes, have not been reported. Meanwhile, the receptor proteins of H_2_S regulating the senescence of plants have not been clearly reported at present. The interaction of H_2_S with other molecules may exert significantly roles in the harvested storage of horticultural products, while its in-depth mechanisms have not been reported in detail until now. In addition, the safety of H_2_S in the food chain, which is important to be considered in the consumption of fruits and vegetables, has also not been reported. Therefore, it is suggested that the future study of H_2_S in postharvest storage should focus on its molecular mechanisms and its interaction with other molecules. Furthermore, the toxicity of its residues in treated fruits and vegetables should be paid more attention to in future studies.

## Author Contributions

WL provided the idea and revised the paper. JH prepared the manuscript. DH and JZ discussed the article structure. HF and BW finished the figures and tables. CW critically evaluated the manuscript. All the authors read and approved the submission of the manuscript.

## Conflict of Interest Statement

The authors declare that the research was conducted in the absence of any commercial or financial relationships that could be construed as a potential conflict of interest.
